# Habitat imaging with intratumoral radiomics for prediction of axillary response after neoadjuvant chemotherapy in breast cancer patients

**DOI:** 10.3389/fmolb.2025.1684809

**Published:** 2025-10-01

**Authors:** Xiaomeng Ji, Bingxin Zhao, Yan Mao, Meng Lv, Yongmei Wang, Xiaohui Su, Zaixian Zhang, Jie Wu, Qi Wang

**Affiliations:** ^1^ Department of Radiation Oncology, The Affiliated Hospital of Qingdao University, Qingdao, Shandong, China; ^2^ Breast Disease Diagnosis and Treatment Center, The Affiliated Hospital of Qingdao University, Qingdao, Shandong, China; ^3^ Department of Radiology, The Affiliated Hospital of Qingdao University, Qingdao, Shandong, China; ^4^ Department of Pathology, The Affiliated Hospital of Qingdao University, Qingdao, Shandong, China

**Keywords:** breast malignancy, habitat region, machine learning, magnetic resonanceimaging, neoadjuvant chemotherapy

## Abstract

**Rationale and objectives:**

Breast cancer remains a leading cause of cancer-related morbidity and mortality globally. This study aimed to develop and validate predictive models for ALN pCR following NAC in breast cancer patients.

**Materials and methods:**

We conducted a retrospective analysis involving 189 patients who were diagnosed with primary breast cancer at the Affiliated Hospital of Qingdao University. Dynamic contrast-enhanced magnetic resonance imaging (DCE-MRI) was utilized to assess the characteristics of the tumors. Tumor segmentation was performed using itk-SNAP software, followed by voxel clustering to identify distinct habitat-derived regions. Logistic regression (LR) and multilayer perceptron (MLP) models were constructed using these features.

**Results:**

The classification model incorporating with habitat-based radiomic features demonstrating superior predictive performance (AUC of 0.88 in training and 0.81 in test for LR). A clinicopathologic signature that includes factors such as age, hormone receptor status, the Ki-67 index, and clinical stage was established, achieving in an AUC of 0.81. To construct a nomogram, we integrated habitat-derived radiomic signature with clinicopathologic signature. This nomogram attained an AUC of 0.92 for the training cohort and 0.89 for the test cohort. Furthermore, calibration and decision curve analyses confirmed the nomogram’s reliability and practical applicability in clinical settings.

**Conclusion:**

In summary, our results indicate that radiomic features extracted from pre-NAC DCE-MRI can improve the predictive accuracy for ALN pCR following NAC in individuals diagnosed with breast cancer. This finding highlights the promise of personalized treatment strategies for individual patients.

## Introduction

Breast cancer is one of the most prevalent malignancies worldwide, presenting major challenges in both diagnosis and therapeutic approaches (Sung et al., 2021). The heterogeneity of breast cancer, with varied biological behaviors and therapeutic responses, highlights the importance of personalized treatment strategies.

Neoadjuvant chemotherapy (NAC) is widely acknowledged as the standard therapeutic strategy in clinical practice for patients with locally advanced or early-stage invasive breast cancer who are candidates for breast-conserving surgery (BCS) (Navarro-Cecilia et al., 2013). Previous research have shown that 20%–40% of breast cancer patients treated with NAC achieve pathologically confirmed clinically negative axillary lymph nodes (ALNs) post-treatment (Kuerer et al., 1999). At present, it is common clinical practice to recommend that most patients with pre-NAC pathologically positive ALN biopsy results undergo axillary lymph node dissection (ALND). However, ALND can lead to significant complications, such as lymphedema, infection, brachial plexus injury, and paresthesia, which can adversely affect patients’ recovery and quality of life. Sentinel lymph node biopsy (SLNB) presents a less invasive option compared to ALND. Nevertheless, the utility of SLNB in patients with positive ALNs undergoing NAC remains controversial. Recent studies have indicated that SLNB-guided axillary surgery is a feasible approach for initially node-positive breast cancer patients who achieve negative ALNs after NAC (Galimberti et al., 2016; Piltin et al., 2020). To improve the accuracy of SLNB, various advanced techniques have been employed. These methods include dual tracer staining, the excision of more than one SLN, and placement positioning pins (Boughey et al., 2015; Boileau et al., 2015; Caudle et al., 2016). These strategies have shown to reduce false-negative rate, but they are not universally applicable to all patients. Therefore, accurately predicting the axillary response to NAC is essential, as it enables the customization of treatment strategies for patients with ALN-positive breast cancer.

Recent advancements in imaging techniques, particularly dynamic contrast-enhanced magnetic resonance imaging (DCE-MRI), have provided new opportunities to characterize tumors and explored their underlying biological mechanisms. DCE-MRI provides valuable insights into the tumor microenvironment and help to identify tumor subregions that may be correlated to clinical characteristics (Hayward et al., 2023). The diagnostic performance of MRI for evaluating ALN status has been significantly improved with the advent of new technologies. These advancements include the use of specialized coils, the optimization of contrast agent protocols, and the integration of coronal plane imaging (Schipper et al., 2015). In addition, preoperative MRI radiomics models, which evaluate both the ALN and tumor regions, have been investigated for their ability to predict ALN status in early-stage breast cancer patients (Yu et al., 2021). However, the performance of MRI for evaluating post-NAC ALN status is considered suboptimal, with an AUC ranging from 0.50 to 0.75 (Cattell et al., 2020; You et al., 2015).

The emergence of radiomics offers new possibilities to extract quantitative patterns from medical images—patterns that are associated with underlying biological characteristics, therapeutic responses, and clinical outcomes. Currently, deep learning radiomics has been utilized to evaluate the efficacy of NAC, especially in predicting pathological complete response (pCR) and overall treatment response. Fu et al. (2024) constructed a deep learning framework utilizing sonographer-conducted axillary ultrasound images to forecast the ALN response to NAC. This model, which integrated both pre- and post-NAC ultrasound data, reached an AUC of 0.95 in the training group and 0.90 in the test group. Our previous investigation established a deep learning model utilizing DCE-MRI conducted before and after NAC to forecast ALN response following NAC treatment (Zhang et al., 2024). The integration of the deep learning model with clinical parameters resulted in the establishment of a predictive radiomic nomogram, which demonstrated exceptional performance, with an AUC of 0.99. Although traditional radiomic models and deep learning models demonstrate significant predictive performance, they are often referred to as “black boxes”. This is because they primarily provide input data and output results, without offering clear interpretability of the intermediate processes or decision-making pathways. As a result, while these models can be highly accurate, their lack of transparency makes it difficult to understand how they reach specific conclusions, which can limit their clinical applicability and acceptance.

The “Habitat” concept in cancer imaging refers to spatial and evironmental features of the tumor microenvironment, arising from intratumoral heterogeneity. An extension of radiomics involves automatic segmentation informative subregions, which are linked to the tumor’s underlying pathophysiology (Napel et al., 2018). Studies have shown tumor habitat analysis can predict treatment responses and clinical outcomes (Shi et al., 2023; Cho et al., 2022; Zhang Y. et al., 2023). In habitat analysis, two common clustering strategies are used: first, calculating voxel relationships to assess heterogeneity; second, extracting radiomic features to enable more detailed clustering and habitat region delineation. Shi et al. (2023) developed intratumoral heterogeneity index utilizing habitat radiomics derived from DCE-MRI to predict the NAC treatment response in breast cancer patients. However, there is currently limited literature on predicting ALN response to NAC using habitat radiomics. Therefore, we investigated the efficacy of a radiomics model—incorporating intratumoral habitat regions from DCE-MRI—in predicting ALN response to NAC among patients with breast cancer.

## Methods and materials

### Patients

The study included 189 patients newly diagnosed with breast cancer at the Affiliated Hospital of Qingdao University in Qingdao, China, between 2021 and 2022. Given the retrospective design of this study, informed consent was not required. The study was approved by the hospital’s ethics committee in accordance with the guidelines of the Declaration of Helsinki. The criteria for participant inclusion were specified as follows: (1) Histopathologically confirmed primary invasive breast cancer; (2) Confirmation of ALN metastases via surgical or biopsy; (3) ALND performed after NAC; (4) Administration of systemic chemotherapy before surgery; (5) Acquisition of adequate DCE-MRI scans before NAC; (6) NAC regimens developed in accordance with NCCN or CSCO guidelines; (7) Availability of comprehensive medical records. On the other hand, the criteria for exclusion included: (1) Patients presenting with distant metastases; (2) Individuals with a prior diagnosis of other malignancies; (3) Patients exhibiting negative ALN findings before the initiation of NAC; (4) Incomplete NAC administration before surgery; (5) Absence of critical clinical information, such as findings related to ALN or specific molecular subtype; (6) Inadequate imaging of the axillary area on breast MRI. Distant metastases were defined and diagnosed in accordance with the NCCN Breast Cancer Guidelines, using multimodality imaging (whole-body ^18^F-FDG PET-CT, chest/abdominal CT, bone scintigraphy) and pathological confirmation for ambiguous lesions when necessary.

### Magnetic resonance acquisition protocol

Prior biopsy, each patient underwent pre-treatment DCE-MRI within one to 2 weeks before NAC. The MRI scans were conducted using a 3.0 T scanner, equipped with either an 8-channel or 16-channel breast coil (Signa HDxt, GE Healthcare), with patients in the prone position. The DCE-MRI procedure included a pre-contrast T1-weighted image and eight subsequent post-contrast T1-weighted images, all with fat saturation. After administering 0.2 mL/kg of gadolinium-DTPA contrast agent intravenously, a 20 mL saline flush was given at a rate of approximately 2 mL/s. Sixty seconds after the gadolinium-DTPA injection started, the first series of post-contrast images was acquired, followed by seven additional scans. The methodology for acquiring MR images has been thoroughly detailed in previous studies (Zhang et al., 2024).

### Tumor segmentation

The N4 bias field correction algorithm was employed for image preprocessing to correct inhomogeneities present in MR images. The detailed parameter settings were as follows: Number of iterations per resolution level: (50, 50, 50, 50); Smoothing kernel: 200 mm per mesh element in each dimension; Mask dilation radius: No additional dilation. These settings refer to the ANTs software guidelines (https://github.com/ANTsX/ANTs/wiki/N4BiasFieldCorrection). The original imaging data was resampled using B-spline interpolation to achieve an isotropic resolution of 3 × 3 × 3 mm^3^. Choosing a 3 mm voxel size was essential to capture radiomic features from numerous voxels and ensure comprehensive collection of local details, despite increasing processing time. Resampling to larger voxel sizes significantly reduces data volume while retaining key information, thereby improving processing efficiency. To reduce inter-image variability and improve the reproducibility of analyses, intensity value histograms from MRI scans were standardized.

Regions of interest (ROIs) were carefully outlined on each slice of the DCE-MRI, focusing on the peak enhancement phase indicated by the time-intensity curve, utilizing itk-SNAP software. During the peak enhancement phase, metastatic ALNs exhibited significant enhancement, while the surrounding stromal tissue showed only slight enhancement. Two radiologists (each with 5 years of breast imaging experience each) performed ROI segmentation in a double-blinded manner—they were unaware of patients’ ALN pCR status, postoperative pathological results, and NAC response. The agreements of the ROIs between different radiologists and within the same radiologist reflect inter- and intra-observer reproducibility, respectively. We used intraclass correlation coefficients (ICCs) to evaluate the consistency of feature extraction among observers. Both inter- and intra-observer reproducibility, as well as radiomic feature extraction, demonstrated substantial agreement, with ICC values exceeding 0.75 for ROIs assessed by the two different radiologists and the same radiologist.

### Habitat imaging and radiomics feature extraction

Twenty-two radiomics features were derived for each voxel within the tumor region to capture local information. A comprehensive list of these features and their definitions is provided in Supplementary Table S1. The 22 voxel-level features include 8 gray-level statistical features, 10 texture features, and 4 dynamic enhancement parameters, with calculation standards consistent with the PyRadiomics documentation (https://pyradiomics.readthedocs.io/en/latest/features.html). Following the feature extraction, voxel clustering was executed using the k-means method. Initial cluster centers were determined via the k-means++ algorithm to avoid random bias, with the maximum iterations set to 1,000 to prevent infinite loops.

This clustering method grouped voxels with similar characteristics, thereby facilitating the identification of distinct habitat regions within the tumor. In order to identify the most suitable number of clusters, the mean Calinski-Harabasz score was employed, evaluating clusters in a range from 2 to 5. The number of clusters corresponding to the highest Calinski-Harabasz score was selected as the optimal clustering configuration. We used the Python package PyRadiomics to extract radiomic features from the intratumoral region and different habitat regions separately.

### Prediction models construction and validation

The dataset was randomly split into training and test sets at an 8:2 ratio. The training cohort was employed for the development of prediction models utilizing the extracted radiomic features. Radiomic features were initially evaluated using the Mann-Whitney U test, with a significance level set at P < 0.05. Subsequently, the Pearson correlation coefficient was utilized to evaluate the relationship between each pair of radiomic features, resulting in the exclusion of features that displayed a correlation coefficient |r| exceeding 0.9. For the training cohort, feature selection was performed using the least absolute shrinkage and selection operator (LASSO) method. The penalty parameter λ was selected by minimizing the mean squared error (MSE) via 5-fold cross-validation.

To develop classification models, both the logistic regression (LR) model and the multilayer perceptron (MLP) were employed. The efficacy of all constructed models was assessed using receiver operator characteristic (ROC) analysis, along with metrics including sensitivity, specificity, positive predictive value (PPV), and negative predictive value (NPV). The practical significance of these machine learning models was explored through decision curve analysis (DCA). A calibration curve was employed to illustrate the relationship between predicted probabilities and observed rates. We developed two model types using (1) habitat-based radiomic features (habitat-based predictive models), and (2) intratumoral-based radiomic features (intratumor predictive model). The robustness of these signatures was subsequently validated using test set.

### Clinicopathologic signature development and validation

A range of clinical parameters, including histopathological features, were assessed for their potential association with ALN pCR. A clinicopathologic signature was developed, incorporating key characteristics such as patient’s age, estrogen receptor (ER)/progesterone receptor (PR)/Her-2 status, Ki-67 index, and clinical T/N staging. Clinical stage specifically refers to the preoperative cTNM stage, which was determined based on preoperative imaging and physical examinations to define cT and cN categories, in strict accordance with the 8th edition of the AJCC Breast Cancer Staging Manual. The postoperative pTNM stage was not used, as the primary objective of this study was to develop a preoperative prediction tool.

We used LR and MLP to develop our classification models. Using the best-performing radiomic signature combined with clinicopathologic signature, we developed a predictive nomogram to facilitate clinical decision-making. This nomogram was constructed via a multivariate regression model, followed by calibration via the Hosmer-Lemeshow test and a calibration curve. In order to evaluate the clinical utility of the nomogram, we plotted a DCA curve, providing insights into its practical applicability in clinical practice.

### Statistical analyses

Statistical analyses were performed using R Studio (version: 2023.12.1) and Python 3.12.2. The chi-squared test and Mann-Whitney U test were used to explore the association between NAC efficacy and various clinical variables. The R packages “pROC,” “rms,” “rmda,” and “generalhoslem” were used to generate the ROC curve, calibration curve, and perform the Hosmer-Lemeshow test. A two-tailed significance threshold of 0.05 was set for the statistical analyses.

## Results

### Patient clinical features

**FIGURE 1:**
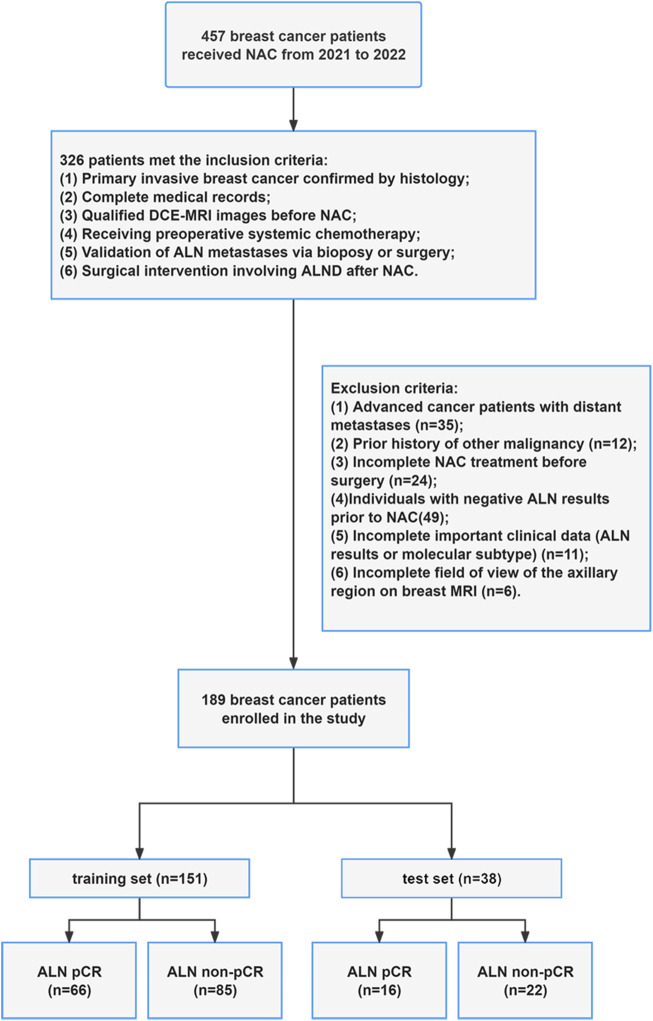
Flow chart of patient enrollment.

A total of 457 MRI examinations were initially collected between 2021 and 2022. Patients were excluded for the following reasons: patients diagnosed with advanced cancer (n = 35), previous history of different malignancies (n = 12), non-completion of NAC (n = 24), incomplete clinical data (n = 11), negative ALN metastases (n = 49), and inadequate axillary region coverage on MRI (n = 6). The patient enrollment flowchart is presented in Figure 1.

A total of 189 patients were included in the study. These patients were randomly divided into training and test cohorts at an 8:2 ratio. The detailed characteristics of all participants are detailed in Table 1. Notably, hormone receptor expression, Her-2 status, and Ki-67 index were significantly associated with ALN pCR after NAC in breast cancer patients. In contrast, no significant differences in age, clinical stage, or menopausal status were observed between patients with ALN pCR and non-pCR. Furthermore, there were no statistically significant differences in clinical parameters (including age, clinical stage, menopausal status, hormone receptor expression, Her-2 status, and Ki-67 index) between the training and test cohorts (see Supplementary Table S2).

**TABLE 1:** The clinical characteristics between ALN non-pCR and pCR patients.

Characteristics	ALN non-pCR	ALN pCR	P-value
Age	107	82	
	48	51	0.12
Menopausal status, n (%)			0.10
Premenopause	65 (60.75%)	40 (48.78%)	
Post-menopause	42 (39.25%)	42 (51.22%)	
ER status, n (%)			<0.05
Negative	29 (27.10%)	47 (57.32%)	
Positive	78 (72.90%)	35 (42.68%)	
PR status, n (%)			<0.05
Negative	40 (37.38%)	54 (65.85%)	
Positive	67 (62.62%)	28 (34.15%)	
Her-2 status, n (%)			<0.05
Negative	85 (79.44%)	23 (28.05%)	
Positive	22 (20.56%)	59 (71.95%)	
Ki-67 index	40 (20, 60)	50 (30, 60)	<0.05
cT stage, n (%)			0.35
T1	7 (6.54%)	5 (6.10%)	
T2	52 (48.60%)	32 (39.03%)	
T3	40 (37.38%)	33 (40.24%)	
T4	8 (7.48%)	12 (14.63%)	
cN stage, n (%)			0.99
N1	92 (85.98%)	70 (85.37%)	
N2	15 (14.02%)	12 (14.63%)	

### Intratumor prediction model development and evaluation

**FIGURE 2:**
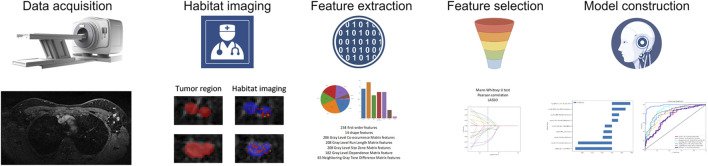
The flowchart of building Habitat-based radiomic signature, clinicopathologic signature, and nomogram.

Figure 2 illustrates the development process of the intratumor prediction signature, habitat-based prediction signatures, clinicopathologic signature, and nomogram. MRI images were manually segmented layer by layer, and features were extracted from these segments. A total of 1,197 features were extracted. After assessing interobserver reproducibility using ICCs, 1,186 radiomic features from the intratumor region were selected for further analysis. Using one-way ANOVA, 6 significant features were identified. For features with a correlation coefficient exceeding 0.9, redundant features were merged into a single representative feature. The 3 features were selected through LASSO regression for further analysis.

We assessed the predictive performance of LR and MLP models for ALN pCR in breast cancer patients treated with NAC. In the training cohort, the AUC for the LR and MLP models were 0.65 (95% confidence interval (CI), 0.57–0.74) and 0.47 (95% CI, 0.37–0.56), respectively. The predictive value of the model was limited, as indicated by sensitivity, specificity, PPV, and NPV(shown in Table 2).

**TABLE 2:** The radiomic model of intratumor and habitat-based models.

Intratumor		Accuracy	AUC	95% CI	Sensitivity	Specificity	PPV	NPV
LR	train	0.65	0.66	0.57–0.74	0.59	0.69	0.60	0.69
LR	test	0.47	0.40	0.21–0.59	0.94	0.14	0.44	0.75
MLP	train	0.46	0.47	0.37–0.56	0.89	0.13	0.44	0.61
MLP	test	0.53	0.55	0.36–0.74	0.75	0.36	0.46	0.67
Habitat1		Accuracy	AUC	95% CI	Sensitivity	Specificity	PPV	NPV
LR	train	0.81	0.88	0.83–0.93	0.79	0.82	0.78	0.83
LR	test	0.79	0.81	0.66–0.96	0.81	0.77	0.72	0.85
MLP	train	0.73	0.82	0.75–0.89	0.86	0.62	0.64	0.86
MLP	test	0.76	0.73	0.55–0.90	0.75	0.77	0.71	0.81
Habitat2		Accuracy	AUC	95% CI	Sensitivity	Specificity	PPV	NPV
LR	train	0.63	0.71	0.62–0.79	0.73	0.55	0.56	0.72
LR	test	0.63	0.55	0.36–0.75	0.25	0.91	0.67	0.63
MLP	train	0.67	0.73	0.65–0.81	0.77	0.57	0.58	0.76
MLP	test	0.53	0.52	0.33–0.72	0.75	0.36	0.46	0.67
Habitat3		Accuracy	AUC	95% CI	Sensitivity	Specificity	PPV	NPV
LR	train	0.56	0.62	0.53–0.71	0.86	0.32	0.50	0.75
LR	test	0.63	0.58	0.39–0.78	0.44	0.77	0.58	0.65
MLP	train	0.56	0.62	0.53–0.71	0.86	0.32	0.50	0.75
MLP	test	0.63	0.58	0.39–0.77	0.44	0.77	0.58	0.65

### Habitat-based predictive models development and evaluation

The Calinski-Harabasz score indicated that 3 was the optimal number of clusters. This analysis revealed 3 distinct subregions in the training cohort, providing valuable insights into inherent data patterns. These identified subregions were subsequently utilized in the test cohort for additional examination.

A total of 1,197 radiomic features were extracted independently from the tumor regions for each habitat. Using one-way ANOVA, 25 features were significant in Habitat 1, 13 in Habitat 2, and 7 in Habitat 3. After Pearson correlation analysis and subsequent LASSO regression, 9 radiomic features from Habitat 1, 7 from Habitat 2, and 2 from Habitat 3 were selected for final model construction.

We constructed classification models using LR and MLP to predict ALN response after NAC based on different habitat subregions. The Habitat 1-based models demonstrated the best performance: the LR model achieved an AUC of 0.88 (95% CI, 0.83–0.93) in the training cohort and 0.81 (95% CI, 0.66–0.96) in the test cohort. Additionally, the MLP reached an AUC of 0.82 (95% CI, 0.75–0.89) in the training group and 0.73 (95% CI, 0.55–0.90) in the test group. In the training group, the Habitat 1-based radiomic model had a sensitivity of 0.79, specificity of 0.82, PPV of 0.78, and NPV of 0.83, while in the test group, these values were 0.81, 0.77, 0.72, and 0.85, respectively.

In contrast, models based on Habitat 2 and Habitat 3 showed limited predictive power. For Habitat 2-based model, the LR and MLP models achieved AUC values of 0.71 and 0.73 in the training cohort, and 0.55 and 0.52 in the test cohort, respectively. For the Habitat 3-based model, both LR and MLP models had an AUC of 0.62 in the training cohort and 0.58 in the test cohort. The detailed results of the habitat-based classifiers are presented in Table 2.

### Development and validation of clinicopathologic signature

The clinicopathologic signature was established utilizing hormone receptor status, clinical tumor stage, and the Ki-67 index. We used various machine learning algorithms to classify patients with ALN pCR after NAC. Both the LR and MLP models achieved an AUC of 0.81 in the training cohort. In the test cohort, both models had an AUC of 0.76, showing comparable predictive performance. Our results showed that the MLP model performed slightly better than the LR model, but the DeLong test indicated no significant difference between them. In the training cohort, the MLP model had an AUC of 0.81 (95% CI, 0.74–0.88), with a sensitivity of 0.42, specificity of 0.91, PPV of 0.78, and NPV of 0.67. In the test cohort, these values were 0.56 (sensitivity), 0.86 (specificity), 0.75 (PPV), and 0.73 (NPV), respectively. Subsequently, the MLP model was utilized to develop the nomogram.

### Nomogram development and validation

**FIGURE 3:**
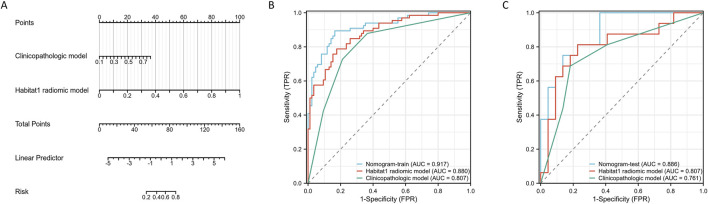
Construction and performance assessment of a nomogram based on Habitat-based radiomic signature and clinicopatholigic signature. **(A)** A nomogram based on the Habitat 1-based radiomic signature and clinicopatholigic signature. **(B)** ROC curves of clinicopatholigic signature, Habitat-based radiomic signature, and nomogram in training group. **(C)** ROC curves of clinicopatholigic signature, Habitat-based radiomic signature, and nomogram in test group.

Given the strong performance of the Habitat 1-based radiomic model, we integrated it with the clinicopathologic signature to develop a nomogram (Figure 3A). Figures 3B,C show the ROC curves of the Habitat 1-based radiomic model, clinicopathologic model, and radiomic nomogram. The nomogram achieved an AUC of 0.92 (95% CI, 0.87–0.96) in the training cohort and 0.89 (95% CI, 0.79–0.99) in the internal test cohort.

**FIGURE 4:**
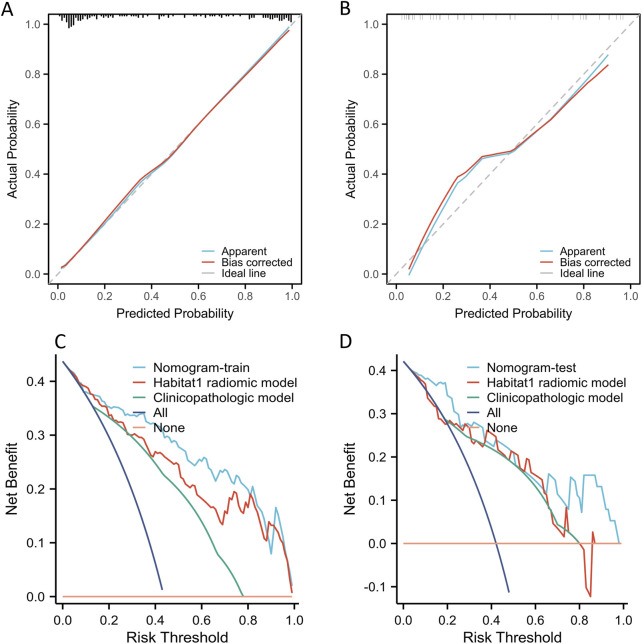
The calibration and DCA curves of Habitat-based radiomic signature and clinicopatholigic signature. **(A)** Calibration curve of Habitat 1-based radiomic signature, clinicopatholigic signature, and nomogram in the training group; **(B)** Calibration curve of Habitat 1-based radiomic signature, clinicopatholigic signature, and nomogram in the test group; **(C)** DCA evaluating the clinical usefulness in the training group; **(D)** DCA evaluating the clinical usefulness in the test group.

The calibration plot showed a wide range of predicted risks, highlighting the model’s ability to distinguish between patients with low and high absolute ALN pCR risks (Figures 4A,B). The Hosmer-Lemeshow test for the nomogram showed χ^2^ = 8.57 (*p* = 0.38) in the training cohort and χ^2^ = 3.84 (*p* = 0.87) in the test cohort. Furthermore, DCA curves indicated that the radiomic nomogram provided greater clinical benefits than the Habitat 1-based radiomic model and the clinicopathologic model (Figures 4C,D).

## Discussion

Breast cancer is a leading cause of morbidity and mortality among women worldwide, characterized by complex biological behavior and significant intertumoral heterogeneity. The advancement of breast cancer is impacted by numerous factors, including tumor microenvironment, hormonal receptor status, and genetic alterations. As our understanding of breast cancer biology evolves, there is an increasing focus on personalized therapies that address these variances. Recent advancements in imaging techniques and radiomics have opened new avenues for non-invasive assessment of tumor characteristics, enabling clinicians to better predict treatment responses and guide personalized treatment. Currently, the majority of researches on using radiomics to predict NAC effectiveness in breast cancer patients primarily focus on predicting pCR of both breast tumor and metastatic lymph nodes (Yu et al., 2023; Peng et al., 2021; Zeng et al., 2023). However, relatively less attention has been paid to predicting ALN pCR specifically.

The purpose of this study was to evaluate the utility of DCE-MRI and radiomic features in predicting the response of ALN to NAC in breast cancer. The study enrolled 189 breast cancer patients with ALN metastases, all of whom underwent axillary surgery after NAC. Postoperative pathological evaluation showed an ALN pCR rate of 43.3%, which is consistent with the results reported in previous studies (Navarro-Cecilia et al., 2013; Cortazar et al., 2014).

Two common clustering approaches are used in habitat analysis: the first directly calculates voxel correlations to assess intratumoral heterogeneity and complexity; the second extracts radiomic features from voxels, leveraging local data for clustering (Cho et al., 2022). The latter approach enables more comprehensive analysis and identification of specific habitat regions. This latter approach facilitates a more comprehensive examination and the identification of specific habitat areas (Yang et al., 2022). In our investigation, we employed the second of these two clustering methods. The Calinski-Harabasz score was used to identify 3 distinct tumor subregions, and radiomic features were extracted independently from each intratumoral habitat region. The Habitat 1-based models demonstrated the highest performance, achieving an AUC of 0.88 in the training cohort and 0.81 in the test cohort for the LR model. In contrast, models based on the whole tumor region, Habitat 2, and Habitat 3 showed limited predictive performance. Given the strong performance of the Habitat 1-based radiomic model, we combined it with the clinicopathologic signature to develop a nomogram. In the training cohort, the nomogram achieved an AUC of 0.92 (95% CI, 0.87–0.96), and in the test cohort, it reached 0.89 (95% CI, 0.79–0.99), reflecting high predictive accuracy in both sets.

A key finding of this study is that habitat-based radiomic models, particularly the Habitat 1-derived LR model, outperformed whole-tumor radiomic models in predicting ALN pCR after NAC in breast cancer patients. This performance gap stems from inherent advantages of habitat analysis that address critical limitations of whole-tumor approaches. First, habitat analysis effectively captures biologically relevant intratumoral heterogeneity. Tumors consist of subregions with distinct properties linked to treatment response, but whole-tumor analysis averages these unique signals, masking key predictive patterns. Second, the whole-tumor region contains overlapping radiomic information, leading to noise and diluted predictive power. The feature selection showed only 3 features were retained from 1,197 whole-tumor features post-LASSO. Habitat analysis, however, groups voxels into homogeneous clusters, yielding non-redundant features—9 were retained from Habitat 1—strengthening the model’s ability to distinguish ALN pCR from non-pCR. Third, habitat analysis offers practical computational efficiency. By segmenting tumors into 3 habitats, it reduces data volume for feature extraction and model training, supporting potential clinical translation without losing key information.

By integrating tumor habitat segmentation and in-depth radiomic feature extraction, we developed predictive models that effectively distinguish between ALN pCR and non-pCR. These results not only highlight the potential of radiomics to improve predictive accuracy but also emphasize the importance of integrating clinical and imaging data in breast cancer treatment decisions.

Radiomic studies on predicting ALN treatment efficacy after NAC in breast cancer patients have primarily focused on breast ultrasound and multisequence MRI. Zhang’s model combines clinical data and ultrasound-derived radiomic characteristics to forecast ALN response among breast cancer patients following NAC (Zhang H. et al., 2023). This integrated model exhibited excellent predictive capabilities, with AUC values of 0.86 in the training group, 0.88 in the validation group, and 0.86 in the external test group. Zhu et al. (2024) studied node-positive breast cancer patients undergoing NAC, extracting radiomic features from the tumor, peritumoral, and ALN regions in pre- and post-NAC MRI scans. The researchers used machine learning approaches to construct a stacking model for predicting ALN response after NAC. This stacking model achieved impressive AUC values of 0.93 in the training cohort, 0.87 in the external validation cohort, and 0.86 in the prospective cohort, respectively. Furthermore, this model showed substantially reduced false-negative rates (14.40%, 20.85%, and 18.18%) when compared to the assessments made by radiologists (40.80%, 50.49%, and 63.64%). The authors also developed an MRI-based deep learning model to predict ALN response to NAC, which achieved favorable performance with an AUC of 0.99 in the training cohort and 0.93 in the test cohort (Zhang et al., 2024). However, traditional and deep learning radiomics models are often considered “black boxes,” as their underlying mechanisms and decision-making processes remain unclear.

Tumors commonly exhibit spatial and morphological variability, with differences in cell density, blood supply, necrosis, and other factors across distinct areas. In cancer imaging, “habitat imaging” refers to the spatial and environmental characteristics of the tumor microenvironment, which arise from intratumoral heterogeneity. Verma et al. (2020) manually segmented habitat regions in glioblastomas using multi-sequence MRI and then evaluated radiomic features to predict tumor progression. Nonetheless, this approach depended heavily on the expertise and discretion of radiologists, which may introduce variability and subjectivity into the results. Shi et al. (2023) developed a predictive model that integrates intratumoral ecological diversity features using pretreatment MRI scans and exhibited excellent performance in forecasting pCR to NAC in breast cancer patients. We followed the methodology of Shi et al. (2023), first calculating the radiomics features for each voxel within the tumor region. Subsequently, voxel clustering was conducted utilizing the k-means algorithm, grouping voxels with similar characteristics to identify distinct habitat regions within the tumor. Following this, radiomic features were extreacted from each habitat subregion, and models were developed using these characteristics. In subsequent exploratory analyses, certain radiomic features from habitat imaging were found to correlate with hormone receptor status and Ki-67 index (see Supplementary Figure S1), but the specific mechanisms underlying these correlations require further investigation. Existing studies have used habitat imaging and transcriptomic data to explore tumor heterogeneity and its impact on treatment outcomes (Su et al., 2023). In future prospective studies, immunohistochemical staining should be performed for markers associated with tumor proliferation, hypoxia, and vascular density. Through spatial registration of these pathological marker-positive regions with Habitat 1 identified on DCE-MRI, the overlap and correlation between Habitat 1 and these biologically distinct zones need to be quantified. Additionally, transcriptomic analysis needs to be integrated into future work. This analytical approach is expected to help uncover the molecular mechanisms underlying Habitat 1 and its predictive role in ALN response to NAC.

Notably, current research on tumor habitat analysis for predicting breast cancer treatment efficacy still relies heavily on traditional radiomics workflows, often depending on manual or semi-automatic segmentation—which may limit reproducibility and scalability. To address this limitation, recent studies have explored automated segmentation of tumor habitat subregions to improve clinical applicability—and such advancements provide valuable insights for refining habitat-based research. For example, Bareja et al. (2024) demonstrated that nnU-Net models achieved robust automated delineation of medulloblastoma tumor habitats across multi-institutional MRI scans. Though focused on medulloblastoma, this work highlights the potential of deep learning-driven automation to replace labor-intensive manual segmentation—a direction that could be extended to breast cancer DCE-MRI to optimize habitat identification in future studies.

The present study represents a significant advancement in the treatment of breast cancer, particularly in predicting ALN response post-NAC. One of the most notable innovations of this study is the integration of habitat imaging and radiomic features extracted from DCE-MRI. This approach enables a deeper understanding of tumor microenvironment by identifying distinct habitat regions within the tumor, an aspect often overlooked in prior studies. In contrast to traditional imaging methods, our technique provides a more detailed characterization of intratumoral heterogeneity, which is essential for developing personalized treatment strategies. Although our study has notable strengths, there are areas that could be improved. First, only one imaging protocol was used for pre-NAC evaluation. While breast DCE-MRI yielded the most definitive results, integrating multiparametric MRI—including diffusion-weighted imaging (DWI) and T2-weighted imaging (T2WI)—could provide more comprehensive and objective information. Additionally, our research lacks external validation, primarily due to the limited number of cohorts that meet our specific inclusion criteria. Although we identified several candidate cohorts, most were excluded because pre-NAC MRI were missing. In the future, independent external validation data will be needed to verify the model’s performance.

In summary, our study developed a predictive nomogram using pre-treatment DCE-MRI images, incorporating habitat imaging and intratumoral radiomics. The model demonstrated robust performance in both the training and test cohorts. This prognostic tool provides a reliable and objective basis for informing individualized surgical decisions regarding ALND in breast cancer patients receiving NAC.

## Data Availability

The raw data supporting the conclusions of this article will be made available by the authors, without undue reservation.

## Supplementary material

SUPPLEMENTARY FIGURE 1 The heatmap illustrating the relationship between certain radiomic features from habitat imaging and the clinicopathologic factors.

## References

[B1] Bareja R. Ismail M. Martin D. Nayate A. Yadav I. Labbad M. (2024). nnU-Net-based segmentation of tumor subcompartments in pediatric medulloblastoma using multiparametric MRI: a multi-institutional study. Radiol. Artif. Intell. 6 (5), e230115. 10.1148/ryai.230115 39166971 PMC11427926

[B2] Boileau J. F. Poirier B. Basik M. Holloway C. M. Gaboury L. Sideris L. (2015). Sentinel node biopsy after neoadjuvant chemotherapy in biopsy-proven node-positive breast cancer: the SN FNAC study. J. Clin. Oncol. 33 (3), 258–264. 10.1200/JCO.2014.55.7827 25452445

[B3] Boughey J. C. Suman V. J. Mittendorf E. A. Ahrendt G. M. Wilke L. G. Taback B. (2015). Factors affecting sentinel lymph node identification rate after neoadjuvant chemotherapy for breast cancer patients enrolled in ACOSOG Z1071 (Alliance). Ann. Surg. 261 (3), 547–552. 10.1097/SLA.0000000000000551 25664534 PMC4324533

[B4] Cattell R. F. Kang J. J. Ren T. Huang P. B. Muttreja A. Dacosta S. (2020). MRI volume changes of axillary lymph nodes as predictor of pathologic complete responses to neoadjuvant chemotherapy in breast cancer. Clin. Breast Cancer 20 (1), 68–79.e1. 10.1016/j.clbc.2019.06.006 31327729

[B5] Caudle A. S. Yang W. T. Krishnamurthy S. Mittendorf E. A. Black D. M. Gilcrease M. Z. (2016). Improved axillary evaluation following neoadjuvant therapy for patients with node-positive breast cancer using selective evaluation of clipped nodes: implementation of targeted axillary dissection. J. Clin. Oncol. 34 (10), 1072–1078. 10.1200/JCO.2015.64.0094 26811528 PMC4933133

[B6] Cho H. H. Kim H. Nam S. Y. Lee J. E. Han B. K. Ko E. Y. (2022). Measurement of perfusion heterogeneity within tumor habitats on magnetic resonance imaging and its association with prognosis in breast cancer patients. Cancers (Basel) 14 (8), 1858. 10.3390/cancers14081858 35454768 PMC9025287

[B7] Cortazar P. Zhang L. Untch M. Mehta K. Costantino J. P. Wolmark N. (2014). Pathological complete response and long-term clinical benefit in breast cancer: the CTNeoBC pooled analysis. Lancet 384 (9938), 164–172. 10.1016/S0140-6736(13)62422-8 24529560

[B8] Fu Y. Lei Y. T. Huang Y. H. Mei F. Wang S. Yan K. (2024). Longitudinal ultrasound-based AI model predicts axillary lymph node response to neoadjuvant chemotherapy in breast cancer: a multicenter study. Eur. Radiol. 34 (11), 7080–7089. 10.1007/s00330-024-10786-5 38724768 PMC11519196

[B9] Galimberti V. Ribeiro F. S. Maisonneuve P. Steccanella F. Vento A. R. Intra M. (2016). Sentinel node biopsy after neoadjuvant treatment in breast cancer: five-year follow-up of patients with clinically node-negative or node-positive disease before treatment. Eur. J. Surg. Oncol. 42 (3), 361–368. 10.1016/j.ejso.2015.11.019 26746091

[B10] Hayward J. H. Linden O. E. Lewin A. A. Weinstein S. P. Bachorik A. E. Balija T. M. (2023). ACR appropriateness Criteria® monitoring response to neoadjuvant systemic therapy for breast cancer: 2022 update. J. Am. Coll. Radiol. 20 (5S), S125–S145. 10.1016/j.jacr.2023.02.016 37236739

[B11] Kuerer H. M. Sahin A. A. Hunt K. K. Newman L. A. Breslin T. M. Ames F. C. (1999). Incidence and impact of documented eradication of breast cancer axillary lymph node metastases before surgery in patients treated with neoadjuvant chemotherapy. Ann. Surg. 230 (1), 72–78. 10.1097/00000658-199907000-00011 10400039 PMC1420847

[B12] Napel S. Mu W. Jardim-Perassi B. V. Aerts H. Gillies R. J. (2018). Quantitative imaging of cancer in the postgenomic era: radio(geno)mics, deep learning, and habitats. Cancer-Am Cancer Soc. 124 (24), 4633–4649. 10.1002/cncr.31630 30383900 PMC6482447

[B13] Navarro-Cecilia J. Duenas-Rodriguez B. Luque-Lopez C. Ramirez-Exposito M. J. Martinez-Ferrol J. Ruiz-Mateas A. (2013). Intraoperative sentinel node biopsy by one-step nucleic acid amplification (OSNA) avoids axillary lymphadenectomy in women with breast cancer treated with neoadjuvant chemotherapy. Eur. J. Surg. Oncol. 39 (8), 873–879. 10.1016/j.ejso.2013.05.002 23711734

[B14] Peng S. Chen L. Tao J. Liu J. Zhu W. Liu H. (2021). Radiomics analysis of multi-phase DCE-MRI in predicting tumor response to neoadjuvant therapy in breast cancer. Diagn. (Basel) 11 (11), 2086. 10.3390/diagnostics11112086 34829433 PMC8625316

[B15] Piltin M. A. Hoskin T. L. Day C. N. Davis J. J. Boughey J. C. (2020). Oncologic outcomes of sentinel lymph node surgery after neoadjuvant chemotherapy for node-positive breast cancer. Ann. Surg. Oncol. 27 (12), 4795–4801. 10.1245/s10434-020-08900-0 32779055

[B16] Schipper R. J. Paiman M. L. Beets-Tan R. G. Nelemans P. J. de Vries B. Heuts E. M. (2015). Diagnostic performance of dedicated axillary T2-and diffusion-weighted MR imaging for nodal staging in breast cancer. Radiology 275 (2), 345–355. 10.1148/radiol.14141167 25513854

[B17] Shi Z. Huang X. Cheng Z. Xu Z. Lin H. Liu C. (2023). MRI-based quantification of intratumoral heterogeneity for predicting treatment response to neoadjuvant chemotherapy in breast cancer. Radiology 308 (1), e222830. 10.1148/radiol.222830 37432083

[B18] Su G. H. Xiao Y. You C. Zheng R. C. Zhao S. Sun S. Y. (2023). Radiogenomic-based multiomic analysis reveals imaging intratumor heterogeneity phenotypes and therapeutic targets. Sci. Adv. 9 (40), eadf0837. 10.1126/sciadv.adf0837 37801493 PMC10558123

[B19] Sung H. Ferlay J. Siegel R. L. Laversanne M. Soerjomataram I. Jemal A. (2021). Global cancer statistics 2020: GLOBOCAN estimates of incidence and mortality worldwide for 36 cancers in 185 countries. CA Cancer J. Clin. 71 (3), 209–249. 10.3322/caac.21660 33538338

[B20] Verma R. Correa R. Hill V. B. Statsevych V. Bera K. Beig N. (2020). Tumor habitat-derived radiomic features at pretreatment MRI that are prognostic for progression-free survival in glioblastoma are associated with key morphologic attributes at histopathologic examination: a feasibility study. Radiol. Artif. Intell. 2 (6), e190168. 10.1148/ryai.2020190168 33330847 PMC7706886

[B21] Yang Y. Han Y. Zhao S. Xiao G. Guo L. Zhang X. (2022). Spatial heterogeneity of edema region uncovers survival-relevant habitat of Glioblastoma. Eur. J. Radiol. 154, 110423. 10.1016/j.ejrad.2022.110423 35777079

[B22] You S. Kang D. K. Jung Y. S. An Y. S. Jeon G. S. Kim T. H. (2015). Evaluation of lymph node status after neoadjuvant chemotherapy in breast cancer patients: comparison of diagnostic performance of ultrasound, MRI and ¹⁸F-FDG PET/CT. Br. J. Radiol. 88 (1052), 20150143. 10.1259/bjr.20150143 26110204 PMC4651396

[B23] Yu Y. He Z. Ouyang J. Tan Y. Chen Y. Gu Y. (2021). Magnetic resonance imaging radiomics predicts preoperative axillary lymph node metastasis to support surgical decisions and is associated with tumor microenvironment in invasive breast cancer: a machine learning, multicenter study. Ebiomedicine 69, 103460. 10.1016/j.ebiom.2021.103460 34233259 PMC8261009

[B24] Yu Y. Wang Z. Wang Q. Su X. Li Z. Wang R. (2023). Radiomic model based on magnetic resonance imaging for predicting pathological complete response after neoadjuvant chemotherapy in breast cancer patients. Front. Oncol. 13, 1249339. 10.3389/fonc.2023.1249339 38357424 PMC10865896

[B25] Zeng Q. Ke M. Zhong L. Zhou Y. Zhu X. He C. (2023). Radiomics based on dynamic contrast-enhanced MRI to early predict pathologic complete response in breast cancer patients treated with neoadjuvant therapy. Acad. Radiol. 30 (8), 1638–1647. 10.1016/j.acra.2022.11.006 36564256

[B26] Zhang Y. Yang C. Sheng R. Dai Y. Zeng M. (2023a). Predicting the recurrence of hepatocellular carcinoma (≤5 cm) after resection surgery with promising risk factors: habitat fraction of tumor and its peritumoral micro-environment. Radiol. Med. 128 (10), 1181–1191. 10.1007/s11547-023-01695-6 37597123

[B27] Zhang H. Cao W. Liu L. Meng Z. Sun N. Meng Y. (2023b). Noninvasive prediction of node-positive breast cancer response to presurgical neoadjuvant chemotherapy therapy based on machine learning of axillary lymph node ultrasound. J. Transl. Med. 21 (1), 337. 10.1186/s12967-023-04201-8 37211604 PMC10201761

[B28] Zhang B. Yu Y. Mao Y. Wang H. Lv M. Su X. (2024). Development of MRI-based deep learning signature for prediction of axillary response after NAC in breast cancer. Acad. Radiol. 31 (3), 800–811. 10.1016/j.acra.2023.10.004 37914627

[B29] Zhu T. Huang Y. H. Li W. Wu C. G. Zhang Y. M. Zheng X. X. (2024). A non-invasive artificial intelligence model for identifying axillary pathological complete response to neoadjuvant chemotherapy in breast cancer: a secondary analysis to multicenter clinical trial. Br. J. Cancer 131 (4), 692–701. 10.1038/s41416-024-02726-3 38918556 PMC11333754

