# Supplementary material for: Habitat imaging with intratumoral radiomics for prediction of axillary response after neoadjuvant chemotherapy in breast cancer patients
Source: Front Mol Biosci. 2025 Oct 1;12:1684809. doi: 10.3389/fmolb.2025.1684809 (PMC12520880; doi:10.3389/fmolb.2025.1684809)
Supplement: Supplementary file 1 [file Table1.docx]

Supplementary Table 1. The detailed radiomics features calculated for each voxel within the tumor region

| Autocorrelation | JointAverage |
| --- | --- |
| ClusterProminence | ClusterShade |
| ClusterTendency | Contrast |
| Correlation | DifferenceAverage |
| DifferenceEntropy | DifferenceVariance |
| JointEnergy | JointEntropy |
| Imc1 | Imc2 |
| Idm | Idmn |
| Id | Idn |
| InverseVariance | MaximumProbability |
| SumEntropy | SumSquares |
