# Supplementary figures and images for: Habitat imaging with intratumoral radiomics for prediction of axillary response after neoadjuvant chemotherapy in breast cancer patients
Source: Front Mol Biosci. 2025 Oct 1;12:1684809. doi: 10.3389/fmolb.2025.1684809 (PMC12520880; doi:10.3389/fmolb.2025.1684809)

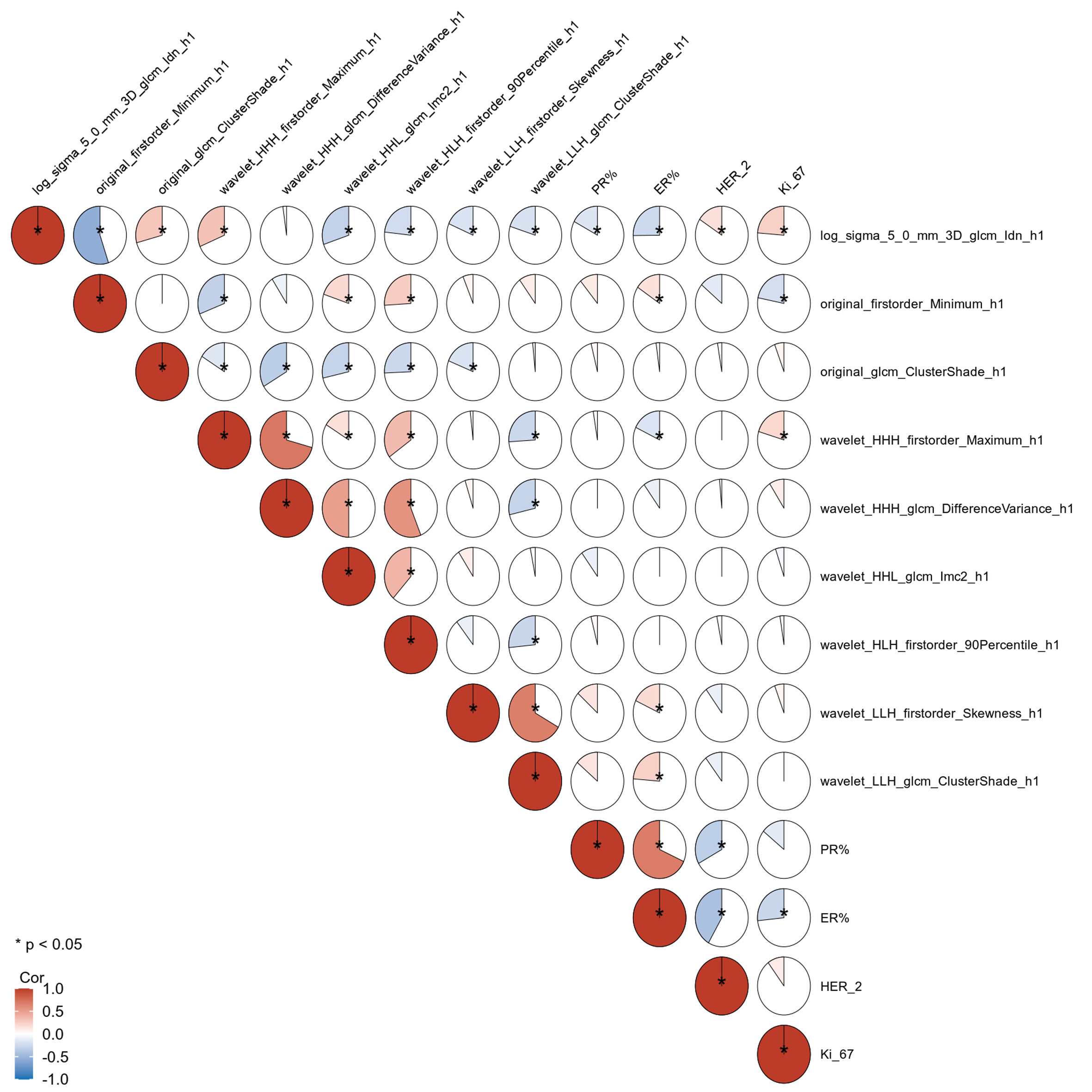

Supplement: Supplementary file 2 [file Image1.tif]
