# Supplementary material for: Habitat imaging with intratumoral radiomics for prediction of axillary response after neoadjuvant chemotherapy in breast cancer patients
Source: Front Mol Biosci. 2025 Oct 1;12:1684809. doi: 10.3389/fmolb.2025.1684809 (PMC12520880; doi:10.3389/fmolb.2025.1684809)
Supplement: Supplementary file 3 [file Table2.docx]

Supplementary Table 2. The clinical characteristics between training and test cohort.

| characteristics | Test cohort | Train cohort | P-value |
| --- | --- | --- | --- |
|  | 38 | 151 |  |
| age | 50 | 49 | 0.85 |
| Menopausal status, n (%) |  |  | 0.26 |
| Premenopause | 18 (47.37%) | 87 (57.62%) |  |
| Post-menopause | 20 (52.63%) | 64 (42.38%) |  |
| ER status, n (%) |  |  | 0.52 |
| Negative | 17 (44.74%) | 59 (39.07%) |  |
| Positive | 21 (55.26%) | 92 (60.93%) |  |
| PR status, n (%) |  |  | 0.65 |
| Negative | 22 (57.89%) | 79 (52.32%) |  |
| Positive | 16 (42.11%) | 72 (47.68%) |  |
| Her-2 status, n (%) |  |  | 0.64 |
| Negative | 23 (60.53%) | 85 (56.29%) |  |
| Positive | 15 (39.47%) | 66 (43.71%) |  |
| Ki-67 index | 40 | 40 | 0.40 |
| cT stage, n (%) |  |  | 0.25 |
| T1 | 2 (5.26%) | 12 (7.95%) |  |
| T2 | 18 (47.37%) | 66 (43.70%) |  |
| T3 | 16 (42.11%) | 55 (36.42%) |  |
| T4 | 2 (5.26%) | 18 (11.92%) |  |
| cN stage, n (%) |  |  | 0.84 |
| N1 | 32 (84.21%) | 130(86.09%) |  |
| N2 | 6 (15.79%) | 21 (13.91%) |  |
| ALN status |  |  | 0.86 |
| ALN non-pCR | 22 (57.89%) | 85 (56.29%) |  |
| ALN pCR | 16 (42.11%) | 66 (43.71%) |  |
